# Maternal Birth Satisfaction Relating to Intraoperative and Early Postpartum Skin-to-Skin Contact with the Neonate During Caesarean Birth: An Integrative Review

**DOI:** 10.3390/nursrep15010028

**Published:** 2025-01-20

**Authors:** Alexandria McCutcheon, Huaqiong Zhou, Mary Steen

**Affiliations:** 1 St John of God Murdoch Hospital, Perth, WA 6150, Australia; 2 School of Nursing, Faculty of Health Sciences, Curtin University, Perth, WA 6102, Australia; 3 Department of Nursing and Midwifery Education and Research (DNAMER), King Edward Memorial Hospital, Women and Newborn Health Service, Perth, WA 6008, Australia

**Keywords:** skin-to-skin, SSC, kangaroo care, intraoperative, caesarean birth, maternal satisfaction, birth satisfaction

## Abstract

**Background**: Mothers and their newborns experiencing caesarean birth often receive delayed or interrupted skin-to-skin care (SSC) despite the intervention being well recognised as beneficial to both mother and baby, with no associated risk for increased morbidity or mortality. Maternal birth satisfaction is recognised as an indicator of quality maternity care; however, most of the research has focused on early intraoperative SSC initiation and breastfeeding outcomes. **Objectives**: To collate and synthesise evidence for maternal satisfaction of intraoperative and early postpartum SSC during and immediately following caesarean birth. To identify timelines of implementation, barriers, and facilitators of SSC. **Methods**: An integrative review was conducted guided by the 5-stage Wittemore and Knalf’s framework. Four electronic databases (CINAHL, Medline, PsycINFO, Web of Science) were searched. Key terms were ‘Caesarean birth’, ‘skin-to-skin care’, ‘maternal satisfaction’. Studies published from 2014 to 5 September 2024 in English language were included. A hand search of potential inclusion articles was also searched to undertake a comprehensive review. The JBI critical appraisal checklist was used to assess the quality of inclusion studies. **Results**: 17 studies met the selection criteria and were included in this review. Intraoperative and early SSC during caesarean birth is associated with positive maternal birth satisfaction and contributes to improved birth experience for mothers with no negative implications. **Conclusions**: Increased access to intraoperative SSC will likely contribute to increased maternal satisfaction and positive birthing experience. Compliance with policy recommendations regarding SSC may improve with access to a flow chart tool identifying expectations of women’s intraoperative and postoperative care for caesarean birth.

## 1. Introduction

Assessing maternal satisfaction of birth experience is an effective indicator of the quality of maternity services [1,2,3]. Maternal birth satisfaction is associated with mothers’ experience of care and may contribute to either positive or negative self-esteem [4,5]. Woman-centred maternity care and services can contribute to a woman’s positive experience of transition to motherhood [4,6]. It has been reported that increasing mothers’ birth satisfaction improves their health outcomes and can be effective for reducing the incidence of postpartum psychological trauma, including post-traumatic stress disorder (PTSD) [5], whilst also contributing to a reduced incidence of postnatal depression and anxiety (PNDA) [7].

Women experiencing caesarean birth have a known decreased incidence of birth satisfaction when compared to their spontaneous vaginal birth counterparts [8]. Caesarean birth represents a large proportion of the birthing population in Australia; in 2021, 118,887 women experienced caesarean birth out of a total of 311,360 births, which equates to 38.2% of the national birthing cohort. The Western Australian cohort experienced a caesarean birth rate of 39.4% (13,478 of 34,201 births), a representation of 1.2% higher than the national cohort [9]. Pregnancy is not pathological—it is a physiological process that has been overtly pathologised by routine medicalisation and intervention in modern healthcare [10]. Medicalisation and high levels of intervention contribute to a higher caesarean birth rate than advocated by WHO, without an associated improvement in maternal and neonatal morbidity and mortality and with significant public health implications [11].

Birth in our community occurs along a continuum from the absence of any intervention, as a completely physiological process, to extensive elective or emergent intervention [12]. With the medicalisation of childbirth, there has been a degree of separation of mother and baby; caesarean birth within a sterile field, clamping and cutting of the umbilical cord, and manual removal of the placenta create a physical detachment of the mother–baby dyad [13,14,15].

Skin-to-skin care of the neonate is a physiologically normal act whereby the mother holds the naked baby in contact with her skin, usually on her chest, in the time immediately following birth. It is biologically appropriate for the newborn to remain near their mother during physiological childbirth and continue to be attached by the umbilical cord and placenta, which remain within the uterus until the third stage of labour is complete [13,14]. It is evident within the research that skin-to-skin contact optimises outcomes for the mother-baby dyad including neonatal thermoregulation and blood glucose regulation, bonding and initiation of breastfeeding [15]. Another study [16] reports a positive correlation between early maternal-neonatal contact and a positive birth experience, but these data are not specific to skin-skin contact during caesarean birth.

Over recent decades, research has presented a specific focus on skin-to-skin care and breastfeeding initiation and duration; however, there can be a vast set of factors that influence initiation of breastfeeding, thus an opportunity exists in which mothers’ perspectives and satisfaction of their birthing experience can be explored. A study [17] also investigated the correlation between intraoperative, skin-to-skin contact and reduced maternal requirement for analgesic and anxiolytic support intraoperatively demonstrating that skin-to-skin contact, likely by release of endorphins and oxytocin, reduces maternal pain and anxiety.

There exists a scope to consider skin-to-skin care from a “what do women want” perspective and look at maternal satisfaction at the forefront of the birthing experience rather than a focus upon morbidity and mortality. This is further reinforced by the theory of Salutogenesis by Antonovsky, an approach to health and well-being as a sense of coherence and successful coping with challenges or interventions [18]. It is critical to emphasise women’s health and well-being at the forefront of maternity care rather than risk and illness to move towards a holistic approach to birth in the hospital setting [19].

An ethnographic study [20] describes the ‘perceived ownership’ within a caesarean birth; the bottom half of the woman ‘is owned’ by the obstetrician, the top half of the woman is owned by the anaesthetist and the baby is owned by the midwife. Any opportunity to address this ownership, autonomy and sovereignty over her birth belongs to the woman [21]; an opportunity to assume her role as mother and take “ownership” of her baby and also the top half of her body that is in physical contact with the baby.

Whilst the benefits of early and uninterrupted skin-to-skin contact for mothers and babies during the transition to extrauterine life are well known and recognised by WHO and in state tertiary hospital policy [2,22,23], standard practice across various healthcare settings does not include consistent implementation of immediate intraoperative skin-to-skin contact. Depending on the clinical setting, site-specific policy and implementation of these policies by the specific care providers present this intraoperative skin-to-skin is facilitated on an ad hoc basis. One study [24] cites the absence of specific policy regarding skin-to-skin contact and individual healthcare providers’ beliefs about the benefits of skin-to-skin as barriers to intraoperative skin-to-skin practice, whilst another [25] considers safety concerns and inadequate staffing levels as barriers.

Skin-to-skin care intraoperatively and immediately following caesarean birth offers an opportunity for a return to a physiological innate process following an intervention in birth rather than further perpetuating the cascade of intervention and separation of mother and baby. The missed opportunity for skin-to-skin contact intraoperatively and immediately following caesarean birth may contribute to decreased overall maternal satisfaction with the birthing experience.

Current research appears to focus of the initiation of SSC and/or breastfeeding and health benefits. There appears to be a lack of literature and research that focuses on maternal satisfaction of intraoperative and early postpartum SSC during and immediately following caesarean birth. Therefore, this review addresses an identified gap in the literature. It is vitally important to identify any evidence that has explored or investigated maternal satisfaction, as this outcome is a key quality care indicator. Knowing how satisfied mothers are will support and guide the implementation of the clinical practice of SSC intraoperative and early postpartum SSC during and immediately following caesarean birth.

This reviewed aimed to collate and synthesise evidence of a relationship between maternal satisfaction of the birthing experience and the implementation of skin-to -skin contact intraoperatively and in the immediate postnatal period for healthy and well women and babies experiencing caesarean birth.

Specific objectives were:

(1) To identify the maternal experience of intraoperative and early skin-to-skin contact, in comparison with standard care (post-operative skin-to-skin) following caesarean birth;

(2) To identify any timeframes for implementation and duration of skin-to-skin contact intraoperatively and immediately following caesarean birth when it has been implemented in the clinical setting;

(3) To identify any reported barriers and facilitators affecting the implementation of skin-to-skin care intraoperatively and immediately following caesarean birth;

(4) To make recommendations for clinical practice based on findings.

This review explored current literature to confirm or refute whether the clinical practice of intraoperative and immediately following caesarean birth skin-to-skin care for women would likely contribute to increased maternal birth satisfaction and therefore reduce risk for postnatal depression and improve longitudinal outcomes for women and their families.

## 2. Materials and Methods

An integrative review was chosen rather than a systematic review to allow the inclusion of studies that utilised a range of methodologies to either explore and/or investigate maternal satisfaction of intraoperative and early postpartum SSC during and immediately following caesarean birth. This review was guided by a 5-stage framework [26]. This framework was used to ensure a rigorous process for an integrative review of the literature, and each of the 5-stages was followed:

### 2.1. Stage 1—Problem Identification

The PICO acronym was utilised for problem identification to effectively communicate the Population, Intervention, Comparison or Context and Outcome [27]. Utilising a systematic four-part PICO statement to accurately determine the clinical question increases the likelihood of a search returning evidence-based results to best inform clinical practice [28].

P—Women giving birth by caesarean section;

I—Intraoperative and early skin-to-skin contact with the neonate;

C—Compared to routine care;

O—Maternal satisfaction with the birth experience.

### 2.2. Stage 2—Literature Search

An initial search of the literature identified key terms for skin-to-skin: skin-to-skin STS, skin-to-skin-care SSC; kangaroo care, caesarean birth: caesarean, c-section, abdominal delivery and maternal satisfaction, satisfaction, birth satisfaction, well-being. An academic librarian was consulted in the process of creating a search strategy for this integrative review, three concepts were identified as “caesarean birth”, “skin-to-skin” and “satisfaction or well-being”. Whilst the PICO acronym was utilised in generating the clinical question [27] utilising all components of PICO in the literature search can reduce number of results generated from the search; therefore, key concepts P-population and I-intervention were used to conduct the search [29].

On 19 April 2024, a comprehensive search of the following databases was conducted: Medline, PsycINFO, CINAHL and Web of Science. This search was then repeated on 5th September 2024. These databases were selected based on their relevance to health sciences and midwifery. Boolean operators and truncation of the key concepts were utilised to conduct the search, with consideration for spelling differences and acronyms. Search terms: caesarean OR cesarean OR “c-section” OR “abdominal deliver*” OR “postcaesarean OR postcesarean OR “post-caesarean” OR post-cesarean” AND “skin to skin” OR Kangaroo care OR SSC OR STS. Limiters were applied to ‘full text available’ and 2014 to 2024 were applied to search results. The timeframe for the literature search start date, 2014, reflects the comprehensive literature review of skin–skin contact after a caesarean section [30]. There were no limitations applied to the study design or methodology.

Inclusion criteria were healthy and well women at term gestation who had a caesarean birth whether elective or non-elective. Studies with participants that were either primiparous or multiparous women were eligible for inclusion. Intervention was required to be intraoperative skin-to-skin or early post-partum skin-to-skin contact following the caesarean birth. Outcome measures were maternal birth satisfaction or positive birth experience. Any results returned where the population studied were clinical staff (nurses or midwives) were ineligible for inclusion.

**Figure 1:**
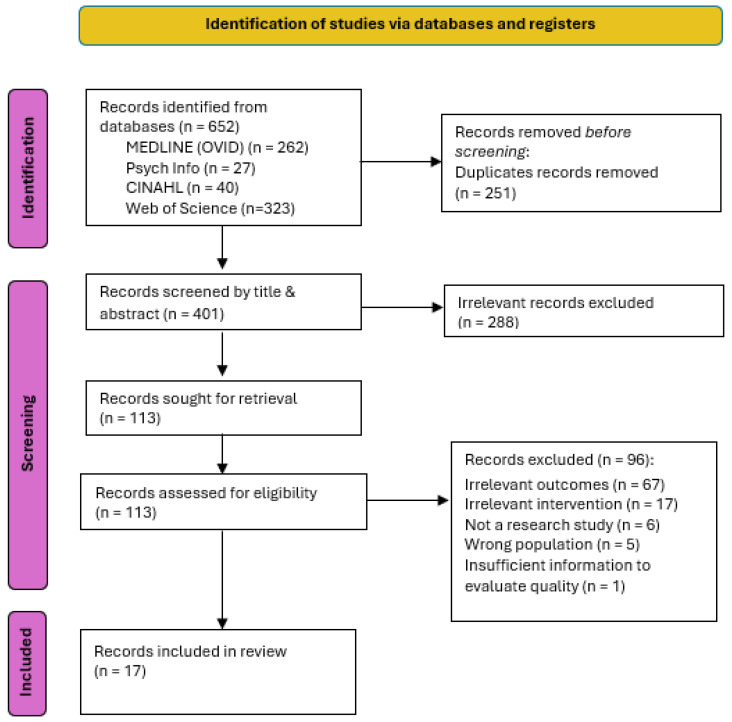
PRISMA flow diagram.

Endnote referencing manager was utilised for managing search results, and the web-based collaboration software programme, Covidence (Version 2) was utilised to screen the results. The first author, AM, screened the initial search of titles, keywords, and abstracts. AM and MS reviewed the full text of potential inclusion studies. HZ was brought in to review two studies that the first two reviewers did not reach consensus. A PRISMA flow diagram was utilised to document the screening and selection process (refers to Figure 1) [31].

### 2.3. Stage 3—Data Evaluation

Potential studies were subject to quality appraisal with the relevant Joanna Briggs Institute [JBI] critical appraisal checklist, JBI levels of evidence also noted (JBI, 2013) [32].

### 2.4. Stage 4—Data Analysis

Data from the articles selected were manually extracted and summarised. Key themes and other pertinent information were identified. Extraction of this information assisted in the process of observing trends in the data and common themes and findings. These data were then collated into a table format for visualisation and ease of interpretation of findings [26]. The information extracted from the review study is presented in Table 1 and Table 2.

### 2.5. Stage 5—Presentation

Tabulation has been utilised to display the information extracted from included articles for ease of review by the reader and interpretation and synthesis of the data. Table 1, Table 2, Table 3 and Table 4; and the information has been utilised to construct a narrative review.

## 3. Results

The initial search of the electronic databases returned 652 records, with 251 duplicate records removed by automation tools. The title and abstract of the remainder of 401 records were screened for relevance, resulting in further exclusion of 288 records. The rest of 113 full text reports were retrieved for review against selection criteria and resulted in 96 exclusions with reasons: irrelevant outcomes (n = 67), irrelevant intervention (n = 17), not a research study (n = 6), wrong population (n = 5), insufficient information in the publication to evaluate (n = 1). Seventeen articles of various methodological approaches met the inclusion criteria (Figure 1).

Table 1 summarises characteristics of included studies. Quantitative articles included one randomised controlled trial [33], two prospective cohort trials [40,43], one cross-sectional study [35], three quasi-experimental studies [24,36,47] and one secondary data analysis [39]. Four qualitative articles utilised a phenomenological approach [34,38,41,46]. Two mixed-method studies were identified [42,45], and three integrative reviews [37,39,46].

Geographical distribution of the results demonstrates seven studies originating from USA, five from Australia, two from Germany, and one each, respectively, from Switzerland, Israel, and Canada. This distribution demonstrates a growing interest in maternal birth satisfaction related to intraoperative SSC in American and Australian research populations.

Table 2 displays the theme and subthemes of main findings extracted from the included studies. All 17 studies had a common theme of increased birth satisfaction or positive birth experience associated with intraoperative SSC. Six studies described a sense of empowerment or control reported by the women receiving intraoperative SSC [34,37,38,41,46,47].

Five studies provided insight into a common theme of increased bonding/mutual care-giving or reciprocal comfort between mother and baby [24,33,38,42,45].

Simultaneously two subthemes emerged from the articles related to decreased incidence of postnatal depression and anxiety [44,45] and a “gentle” more “natural” caesarean birth experience for women [33,35,37,46,47].

One study [35] explored, in addition to early SSC, the constant visualisation of the birth by the woman utilising a transparent drape; whilst intraoperative SSC is associated with increased maternal satisfaction and a positive birth experience, there was no associated advantage or disadvantage to the addition of a transparent drape. However, these researchers reported that there may be scope to consider the utilisation of a transparent drape and potential benefits in circumstances where early skin-to-skin may not be possible; for example, pre-term birth with associated risk factors [35].

Timelines for the implementation of SSC vary across all studies, ranging from immediately post-birth after the cord is clamped and cut [24,33,35,36,38,40] to within 5 min of birth [44,45,47] and within 2 h of birth [42]. There is limited evidence provided by the included studies of the duration of skin-to-skin contact, with only two studies documenting at least 1 h duration [33,38] others “until end of surgery” [35,47], an intervention group of approximately 300 min duration [24], and greater than 20 min duration [44]. These timeframes are reported in Table 3.

Identified barriers to implementation of intraoperative SSC included the lack of staff [30,37], reluctance for practice change [30], healthcare practitioner concerns regarding neonatal thermoregulation, and the need for a safe method of transfer of mother and baby from the operating table to the postoperative bed [45]. A listed facilitator to implementation of SSC was the provision of adequate neonatal monitoring equipment [24,39]. Some strategies for the implementation of SSC intraoperatively included effective communication with all key stakeholders, education for staff and women about the importance of SSC, and having designated staff for the role of providing SSC [47]. A study [39] recommended accessing any published implementation strategies to utilise as a framework for implementation in a clinical setting. Another study [24] recommended utilising the Healthy Children’s Skin-to-Skin Algorithm for Immediate, Continuous, Uninterrupted SSC contact, for identifying compliance with best practice skin-to-skin. These strategies, barriers, and facilitators are presented in Table 4.

## 4. Discussion

Previous researchers [30] identified a significant need to explore further the impact and implementation of intraoperative and early skin-to-skin care following caesarean birth. In the decade since, there has been some exploration into the impact of skin-to-skin practice and how to effectively implement this intervention in theatre and postanaesthetic recovery units. As far as the authors are aware, this is the first integrative review that specifically explores the outcome of maternal satisfaction associated with SSC during and immediately post-caesarean birth.

### 4.1. Maternal Satisfaction

The USA and Australia appear to be at the forefront of exploring and investigating the impact of SSC intraoperatively. The theme of maternal satisfaction emerged from all 17 articles in this review; all findings indicated that there is a positive impact of SSC during and post-caesarean birth and that SSC is well received by the caesarean birth population. Women experiencing SSC intraoperatively reported increased bonding, sense of control, and increased birth satisfaction.

Maternal satisfaction is an accepted identifier of quality maternity services. Considering maternal satisfaction at the forefront of decision making in maternity care has several implications: it is a reminder that pregnancy is not pathological, women are well and can remain well during pregnancy, birth, and postpartum, and that babies and mothers are best placed when considered as a dyad rather than separate entities [48].

If maternity service providers seek to promote or increase maternal satisfaction with regard to birth experience, this can be achieved by initiating evidence-based best practice of SSC and enabling opportunities for longer duration.

This implementation of SSC clinical practice will contribute to increased birth satisfaction and self-efficacy and decreased risk for postnatal depression and anxiety. Health services are consistently impacted by healthcare budgets and cost-based implications for practice. SSC is a free intervention that requires minimal, if any, additional resources to implement and provides women birthing by caesarean an opportunity to claim “ownership over their baby” and commence their transition into motherhood.

The term Charité caesarean birth CCB [33] refers to the modified caesarean birth protocol implemented at Charité Hospital Berlin, whereby women are provided an opportunity to experience their birth in a warmed theatre with dimmed lighting, visualise the birth of their baby by having the surgical drape dropped, commence immediate intraoperative skin-to-skin and have their support person cut the umbilical cord. This protocol is reflected similarly by the ‘extended-gentle caesarean’ birth [35] at Bern university hospital, Switzerland, whereby direct visualisation of the birth is facilitated by a transparent drape, rather than dropping the drape, and the remainder of the post-birth interventions are the same as CCB. ‘Gentle-caesarean’ encompasses all intrapartum care of ‘extended-gentle-caesarean’ without the direct visualisation of the birth [35]. Whilst it is apparent in the findings that the most significant of the interventions for maternal satisfaction in both settings is the early intraoperative SSC, the additional opportunity for direct visualisation of the birth is generally acceptable to women, should be offered in addition to intraoperative skin-to-skin and may be a suitable option to consider for the circumstances when intraoperative SSC is not possible [33,35].

### 4.2. Timeframes for Initiation and Duration

As presented in Table 3, there are various descriptions of timelines for implementation of SSC during caesarean birth, with no set parameters to define time of onset, duration, or rationale for interruption of SSC. World Health Organisation [49] cites a systematic review [15] and defines immediate SSC when the neonate is skin-to-skin with the mother within the first ten minutes after birth and then also considers initiation within the first twenty-four hours to be early skin-to-skin. However, one study [50] states that the 9 stages of normal instinctive newborn behaviour are crucial to the first hour after birth and best facilitated with immediate (post-birth without delay) skin-to-skin contact with the mother. Immediate intraoperative SSC without any delay was able to be facilitated in several research groups [24,33,35,36,40,44] whilst initiation within five minutes was noted with others [44,45,47]. Based on this observed capacity to provide intraoperative SSC within 5 min of birth, it appears that for future research, the following definitions of SSC would be helpful to guide health professionals to initiate SSC as below:

Optimal intraoperative SSC: Initiated within 5 min of birth

Early intraoperative SSC: Initiated between 5 and 10 min post birth

Delayed intraoperative SSC: Initiated between 10 min post birth and prior to completion of the caesarean procedure.

Post-operative SSC: Initiated after completion of the caesarean birth (likely initiated in Post-Anaesthetic Care Unit [PACU]).

However, the continuum of SSC remains a challenge as the included studies reported a range of time period.

### 4.3. Barriers and Facilitators

Access to adequate monitoring equipment to effectively observe neonatal transition is outlined as a significant barrier to implementation [24,39]. Pulse oximeters are readily available for use in Australian maternity settings, and application during skin-to-skin for monitoring in the immediate 1–2 h after birth is standard practice [51]. Pulse oximeters are non-invasive, can be easily implemented and attached during SSC, and effectively utilised to reduce healthcare provider concerns regarding neonatal oxygenation; their use is also acceptable to women and their families [52].

This review has identified barriers to implementation of SSC, including staffing issues and reluctance to change practice. The authors recommend that health professionals’ continual professional development education and training sessions relating to initiation of SSC and breastfeeding emphasise the importance of the health benefits for both the mother and baby. The implementation of intraoperative and early postpartum SSC during and immediately following caesarean birth is not a time-consuming task, and guidelines support this clinical practice. The inclusion of evidence-based statements, i.e., intraoperative and early postpartum SSC during caesarean birth, is associated with positive maternal birth satisfaction and contributes to improved birth experience for women with no negative implications that should be emphasised. Additionally, evidence is emerging to show that fathers can also give SSC during the intraoperative and early postpartum period if for some reason the mother is unable to, and the implementation of this clinical practice is becoming more common [53].

The inclusion and easy access to an SSC flow chart tool within operating and recovery clinical settings may enhance the implementation of SSC intraoperatively and postoperatively. Undertaking a clinical audit and sharing findings may assist SSC to be routinely adopted as best practice.

### 4.4. Recommendations for Clinical Practice

To successfully implement a policy that facilitates, encourages, and provides scope for optimal intraoperative skin-to-skin care, a transparent and easy-to-follow pathway to initiation would provide clarity and reduce the issues with compliance with best practice.

The introduction of a flow diagram that communicates expectations of women’s experience intraoperatively would likely facilitate increased provision of routine skin-to-skin care for neonates following caesarean birth. Flow diagrams are widely accepted in healthcare as effective communication tools that provide all health staff members of a multidisciplinary team and the patient with an understanding of expectations of care [54].

### 4.5. Breastfeeding Outcomes

It is widely accepted that early skin-to-skin contact with the newborn facilitates improved breastfeeding success [55]. Facilitating early and uninterrupted skin-to-skin contact as soon as possible after birth is recommended by the WHO 10 steps to successful breastfeeding [49]. Six studies found intraoperative SSC to have a positive impact on breastfeeding experience [24,33,36,41,42,45]. Two of these studies found that SSC facilitated a shorter time to initiation of breastfeeding [36,45]; in particular, one study [36] also reported an increased overall duration of breastfeeding and an increased rate of exclusive breastfeeding. Participants in another study were able to compare their previous experience of breastfeeding initiation without early SSC and found that the early intraoperative SSC facilitated better breastfeeding experiences [41].

### 4.6. Recommendations for Future Research

A standard set of parameters for the measurement of the implementation of intraoperative SSC would be beneficial such that findings can be easily compared across different sites and settings. Utilising the definitions of SSC listed above would assist researchers in defining timelines of the implementation/onset of SSC.

There is also scope for further research to specifically address the safe transfer of the mother and baby dyad from the operating theatre OT bed to the postop recovery bed, as this appears to be a noted reason for interruption of the SSC in current clinical guidelines [51]. This identified need for further investigation has previously been reported [45].

### 4.7. Strengths and Limitations

A strength of this review is that it included a range of studies and adopted a rigorous process to conduct the selection and critical analysis of the data. The review was conducted by three researchers, one an early-career researcher, one mid-career researcher, and one senior-career researcher, but as with all types of research, there is the risk of researcher bias, despite the utilisation of the Whittmore and Knafl’s 5-stage framework and relevant JBI critical appraisal tools. Additionally, a limitation was that the search results were limited to the English language, and there is a potential risk for language and publication bias. The included research studies were all conducted in high-resource countries, and whilst the findings demonstrate increased maternal satisfaction when experiencing SSC intraoperatively and early postoperatively, this outcome may not be generalisable to low- or middle-resource countries. The practical implications in operating and recovery clinical settings need to be considered. Initiation of intraoperative and early postpartum SSC is not a time-consuming task for a health professional and can be spontaneously undertaken whilst the mother is being cared for during the intraoperative and early post-partum period.

## 5. Conclusions

Increased access to intraoperative SSC for women experiencing caesarean birth will likely contribute to increased maternal satisfaction and a positive birthing experience. Compliance with policy guideline recommendations regarding SSC may be increased with a clear clinical care pathway and access to a flow chart tool identifying expectations of patient intraoperative and immediate postoperative care for caesarean birth. This clinical pathway and tool may assist with increased understanding of the expectations of policy-specific intraoperative care for women undergoing caesarean birth who are well and who have babies born vigorous at birth. Advocating for women to receive intraoperative and uninterrupted SSC during and after caesarean birth may be easier for midwives who can utilise the flow chart for decision making as a tool for collaborating with the multidisciplinary team. Further research into safe bed-to-bed transfer of the mother–baby dyad from the operating table to the recovery bed is needed to address an identified safety concerns related to this transfer and likely unnecessary interruption of the mother and baby dyad during this time.

## Figures and Tables

**Table 1:** Characteristics and main findings of 17 included studies.

Reference	Participants	Study Design	Findings	JBI Level of Evidence	Strengths and Limitations	Recommendations for practice
[33] Germany	205 women having elective CS birth at term, low risk pregnancy, Spinal anaesthetic (intervention n = 102 control n = 103)	RCT study	Birth satisfaction a more positive experience for intervention group. No difference in morbidity or mortality	1c	Did not include patients undergoing non-elective CS birth	Implementation of skin-to-skin “Charitè” caesarean birth should be made available to all non-emergent CS birth. Research should be conducted to determine outcomes for emergency CS birth
[34] USA	13 women who experienced skin-to-skin during caesarean in the past 10 years	Qualitative descriptive study	Women’s experiences of skin-to-skin increased birth satisfaction: up to 32%	3e	Limitation of social media recruitment, may not be representative of greater population	Skin-to-skin care in cs birth should be policy based and offered to all women as routine care
[35] Switzerland	193 women 154 gentle caesarean birth 39 extended gentle caesarean birth (transparent drape)	Cross-sectional study	More control and participation	4b	Strength: Salmon’s Item List German (SIL-Ger) standardised tool utilised to assess satisfaction	Both intraoperative skin-to-skin and transparent drape should be offered to all women if there are no contraindications
[36] USA	41 women (Experimental n = 25, control n = 16)	Quasi-experimental study	Participants who had immediate SSC in CS birth had earlier BF initiation, longer durations of BF, higher rate of exclusive BF and maternal satisfaction.	2c	Limited discussion/data availability in publication	SSC in CS birth is considered beneficial for all mothers and newborns and should be implemented in all environments.
[24] USA	40 women (Group 1 n = 20) (Group 2 n = 20)	Quasi-experimental study	Intraoperative STS is a simple intervention	2c	Small sample size	Health professionals have ethical responsibility to implement immediate and uninterrupted STS and minimise maternal neonatal separation as standard care.
[37] Australia	13 papers, Quantitative n = 6 Qualitative n = 5 Mixed methods n = 2	Integrative Review	Positive correlation between immediate skin-to-skin and positive birth experience	2b	Lack of standardised definition of STS for CS birth, onset and duration. Small samples of the papers	Women want and benefit from SSC. Health professionals should recognise their role in facilitating and advocating for this practice. SSC should be standard care unless medical need for separation. Further research regarding women’s experience of separation at birth needed.
[38] USA	11 women at term gestation having elective CS birth with immediate SSC	Qualitative Ethnography	Intraoperative SSC improves maternal satisfaction, bonding and provides a sense of empowerment and control	3e	Under-representation of primigravida women	Prioritise education of women on the use of SSC during CS. Adv Practice RNs can influence policy on SSC during OT.
[39] USA	3 Qualitative and 10 Quantitative studies	Integrative review	Maternal stress levels reduced, increased comfort, oxytocin. Strong maternal desire to hold and know their baby immediately after birth	2b	Limitations: Small number of articles with wide range of outcomes.	All stable mothers should receive intraoperative skin-to-skin. Appropriate monitoring equipment, education, access to resources of other implementation strategies.
[40] Israel	1833 pregnant women recruited, singleton pregnancy, less than 24 w gestation	Prospective cohort study	Strong association between SSC and birth satisfaction, especially significant for women having cs birth.	3e	Global Birth Satisfaction Tool Limitations: STS assessed across all modes of birth-comparison by mode of birth	SSC is beneficial and improves birth satisfaction for all modes of birth, there is significant increase in CS birth satisfaction with SSC. All women for all modes of birth would benefit from STS.
[41] Canada	10 women with previous CS birth without SSC, booked for Elective repeat CS birth at term, interviewed 1–19 mo postpartum	Qualitative phenomenology	SSC at CS birth is important to women, can improve outcomes for women and infants.	3e	Limitations: Previous CS birth may contribute to findings/confounding factor.	Widespread implementation will require cultural change but is possible.
[42] USA	6 multigravida women booked for repeat CS at term (No SSC at first CS)	Multi-Method Qualitative Descriptive Quasi-experimental study	Reciprocal maternal neonatal comfort, Increased satisfaction compared to previous birth, Felt “Natural”	2d	Strength: Qualitative evaluation, Data saturation achieved. Limitation: No formal survey tool utilised	Delaying non-emergent interventions to facilitate skin-to-skin care may contribute to reluctance of clinicians. Ensure emphasis on improved outcomes for women at the forefront.
[43] Germany	110 women	Prospective cohort study	Women who received “Charitè CS birth” were significantly more satisfied with their birth. satisfaction compared to conventional CS No significant difference in adverse outcomes	3c	Strength Salmon’s Item List German SIL-Ger tool utilis ed.	“Charitè CS birth” (CCB) can be achieved safely outside of emergency situations for planned and unplanned cs birth to improve women’s birth satisfaction. CCB should be standard care for medically necessary CS birth but should not be utilised to increase the rate of ELUSCS.
[44] Australia	Sample of 5840 women who had a live singleton or multiple birth with 30.4% response rate	Secondary data analysis and survey	STS that commenced within 5 min resulted in increased satisfaction		Strengths: Large sample, large dose-response. Limitations: Low response rate, timing of data collection, recall bias.	There is need to further assess the impact of SSC during caesarean birth separately to other modes of birth to effectively explore this intervention.
[45] Australia	(102) 51 SSC during elective CS vs. 51 without SSC during CS birth. >37/40, singleton pregnancy, ELUSCS under spinal, and plan to BF	Quasi-experimental design with qualitative component	SSC during CS birth reduced the time of first feed, increased exclusive BF on DC, Improved bond c newborn, Positive birth experience, reduced risks and interventions, improved satisfaction c care	2c	Continuity of Midwife care may be a confounding factor.	SSC at CB is evidence-based care and is holistically beneficial to mothers and newborns. Recommendation that all health practitioners advocate to provide STS in OT.
[30] Australia	7 papers	Integrative Review	Comprehensive literature search of evidence regarding to immediate and early skin-to-skin CS birth	2b	Small samples of quantitative studies, differences in interpretation of SSC.	Skin-to-skin can be provided safely and immediately. More research to investigate facilitators, barriers, outcomes and BF. Collaboration between healthcare professionals is necessary to achieve STS in OT.
[46] Australia	21 women having CS birth Multigravida women with previous CS birth booked for routine CS.	Qualitative descriptive study	Women want to remain with their baby, have skin-to-skin contact and BF.	2d	Small cohort and only 1 hospital	Health professionals and institutions should recognise the importance of advocating for what women want including encouraging continuous maternal-infant contact and skin-to-skin contact.
[47] USA	583 women: 46 women having SSC in OT during repeat CS assessed for satisfaction. (Simultaneous assessment 60 women having STS assessed for pain perception	Quasi-experimental study	Increased satisfaction with skin-to-skin in OT compared to no SSC	2d	Limitations: Staff participation/adherence to protocol related to confidence/safety concerns.	Communication and education are key to implementing SSC in OT. A designated Nurse for skin-to-skin (appropriate staffing) should be considered.

**Table 2:** Key themes and sub-themes by reference.

Reference	[33]	[34]	[35]	[36]	[24]	[37]	[38]	[39]	[40]	[41]	[42]	[43]	[44]	[45]	[30]	[46]	[47]
Country	Germany	USA	Switzerland	USA	USA	Australia	USA	USA	Israel	Canada	USA	Germany	Australia	Australia	Australia	Australia	USA
Themes																	
Positive Birth/ Birth Satisfaction	X	X	X	X	X	X	X	X	X	X	X	X	X	X	X	X	X
Empowerment/ Sense of Control									X	X							X
Bonding/Mutual caregiving	X				X					X							
Sub-Themes																	
Decreased postnatal anxiety and depression								X						X			
Gentle/ More “natural” caesarean birth	X			X			X					X					X

X—shows that this study reported these themes and sub-themes.

**Table 3:** Initiation, duration, and interruption of SSC by reference.

References	[33]	[35]	[36]	[24]	[40]	[42]	[43]	[44]	[45]	[47]
Initiation of skin-to-skin time	Immediate Post clamp & cut cord	Immediate Post clamp and cut cord	Immediate	G1: Immediate G2: 46 min post birth	Immediate /At birth	Within 2 h	Immediate	49% Cs birth SSC within 5 min	Within 5 min	Within 5 min
Duration of skin-to-skin	At least 1 h.			G1: 300 m G2: 120 m			Until end of surgery	33% held their baby >20 min		Completion of surgery
Reasons for interruption				Routine cares		Routine Cares				

**Table 4:** Implementation, barriers, and facilitators by reference.

Reference	Implementation Strategy
[47]	Effective communication Education for staff and patients Designated RN for SSC in OT
[39]	Accessing published implementation strategies prior to implementation of intraoperative SSC programme.
[24]	Utilising the Healthy Children’s Skin-to-skin Algorithm will facilitate decision making as to which neonates will likely receive SSC.
**Barriers to Implementation**	
[46]	Staff reluctance for practice change, Lack of staff to facilitate SSC
[37]	Inadequate resources, lack of staff education about the benefits of SSC, Hospital practice or policy limitations, workplace culture.
[45]	A safe method of transfer of mother and baby from the OT table to the Postnatal bed needs to be identified. Concerns regarding neonatal thermoregulation.
**Facilitators to Implementation**	
[24,39]	Access to adequate monitoring equipment for neonatal oxygen saturation.

## Guidelines and Standards Statement

Whittmore and Knafl’s framework and JBI tools were used to conduct and report this integrated review.
